# Analysis of Tortuosity in Compacts of Ternary Mixtures of Spherical Particles

**DOI:** 10.3390/ma13204487

**Published:** 2020-10-10

**Authors:** Assem Zharbossyn, Zhazira Berkinova, Aidana Boribayeva, Assiya Yermukhambetova, Boris Golman

**Affiliations:** 1 Department of Chemical and Materials Engineering, School of Engineering and Digital Sciences, Nazarbayev University, Nur-Sultan 010000, Kazakhstan; assem.zharbossyn@nu.edu.kz (A.Z.); zhazira.berkinova@nu.edu.kz (Z.B.); aidana.boribayeva@nu.edu.kz (A.B.); boris.golman@nu.edu.kz (B.G.); 2 National Laboratory Astana, Nazarbayev University, Nur-Sultan 010000, Kazakhstan

**Keywords:** tortuosity, particle compact, ternary mixtures of spherical particles, radical Voronoi tessellation, Dijkstra’s shortest path algorithm, discrete element method

## Abstract

Herein, an approach is proposed to analyze the tortuosity of porous electrodes using the radical Voronoi tessellation. For this purpose, a series of particle compacts geometrically similar to the actual porous electrode were generated using discrete element method; the radical Voronoi tessellation was constructed for each compact to characterize the structural properties; the tortuosity of compact porous structure was simulated by applying the Dijkstra’s shortest path algorithm on radical Voronoi tessellation. Finally, the relationships were established between the tortuosity and the composition of the ternary particle mixture, and between the tortuosity and the radical Voronoi cell parameters. The following correlations between tortuosity values and radical Voronoi cell parameters were found: larger faces and longer edges of radical Voronoi cell leads to the increased fraction of larger values of tortuosity in the distribution, while smaller faces and shorter edges of radical Voronoi cell contribute to the increased fraction of smaller tortuosity values, being the tortuosity values more uniform with narrower distribution. Thus, the compacts with enhanced diffusion properties are expected to be obtained by packing particle mixtures with high volume fraction of small and medium particles. These results will help to design the well-packed particle compacts having improved diffusion properties for various applications including porous electrodes.

## 1. Introduction

The high demand for clean energy and rapid technological development of mobile electrical applications require efficient and environmentally friendly energy storage and conversion systems [1]. Advanced batteries, such as lithium ion or sodium ion batteries, are of great interest owing to their high energy density, environmental friendliness, prolonged cycle life, lightness and generally higher safety compare to conventional energy storage systems [2]. Thus, advanced batteries have been widely used in many applications, including electric vehicles and large-scale energy storage. In the past years, a remarkable progress in research has been made in batteries, especially on the materials’ and cell’s level. Invention of new and improvement of current electrodes materials [3,4], development of various additives and nanocomposites [5,6,7] have significantly enhanced the battery performance over the last decade. However, to meet the increasing criteria for lightweight, energy efficient and fast charging energy systems considerable research and development efforts are still needed and ongoing to improve the available devices and to innovate new concepts, in particular, new electrode materials as they are the main components in the electron and ion transport processes.

Battery electrodes hold the key to energy and power density [8] and their structure alongside the material properties plays a critical role in improving batteries’ performance. Electrodes are mainly manufactured in the form of porous compacts in order to maximize the surface area and to provide percolating paths that can enhance access of ions to the electrode surface and boost the charge storage capacity at the electrode/electrolyte interface while maintaining sufficient mechanical integrity. Thus, recently, the significance of the microstructural architecture of the electrode materials has also been recognized [9,10]. It was demonstrated that the relationships between geometrical (pore size distribution, layer thickness), morphological (porosity, tortuosity) properties of compact, and powder mixture properties (particle size distribution, particle shape) affect the performance of the electrode and the device in general.

In order to understand the effect of these properties on the electrochemical performance of the electrode, considerable efforts have been made both experimentally [11,12,13] and via modeling approaches [14,15]. Experimental approach is more realistic and provides more information on the device performance; however, since it is based on trial-and-error, it is time and resources consuming and can be expensive in particular on the battery pack-scale, whereas numerical modeling approach allows to investigate the effect of microstructure topology on its properties systematically, and thus to identify the optimum one.

In the literature, several approaches on modeling of electrochemical energy storage devices have been established, porous electrode model first developed by Doyle & Newman [16] and its modified versions by a number of research groups [17,18,19,20], and several numerical methods for solving model equations have been proposed based on finite element method [21,22] and Newton-Krylov algorithms [23]. However, the complex electrodes microstructure in these models is over simplified and geometrical features often not taking into account, the electrode microstructure assumed to be homogenous, transport and flow related properties are generalized and treated as continuum.

Recent advances in understanding and modeling of microstructure evolution have followed directly from the emergence of new experimental techniques and developments in computational models. It is now possible to digitize images of real 3D microstructures using algorithms for grain generation and phase assignment. The real 3D images are obtained from X-ray computed tomography (CT) [24] and focused ion beam scanning electron microscopy (FIB-SEM) slice [25]. Although these methods are valuable, they require large-scale equipment; they are costly and time consuming to directly measure numerous cell combinations. Thus, in few recent studies, the discrete element method (DEM) was applied to generate electrode’s packings and mimic the porous electrodes [26]. DEM allows considering granular nature of the electrodes and has become a very powerful numerical tool to characterize the mechanical behavior of granular materials in different fields. This approach also provides an opportunity to capture specific morphological and topological properties of the microstructure and conduct parametric studies in order to evaluate the impact of the individual microstructure characteristic, and to evaluate the pores network. The DEM obtained microstructures were compared to the real systems in a number of studies. It was demonstrated that it is capable of reproducing certain features of the microstructure, although with some approximations on the particle shape and limited particle size distribution. Sangrós et al. [27] calibrated the bond stiffness of the generated DEM packings and the graphite anode showing a good agreement on the force-displacement curves, and demonstrated the possibility of using DEM for mechanical studies of electrode coatings. Delaney et al. [28] developed a ‘virtual laboratory’ platform, where the DEM reproduction and CT images were combined and enabled to obtain multiple mechanical properties of the granular matter. Yan et al. [29] considered the applicability of the DEM approach for simulating the sintering process of the fuel cell electrode, compared the tortuosity values of the virtual electrode structure and the FIB-SEM image. The results of this study demonstrated a great potential of the DEM simulations in generating realistic initial microstructures.

The performance of the electrochemical device is strongly affected by the transport properties of the conducting networks. The key parameters to identify the conducting networks affecting the mass transport in porous media are tortuosity and porosity. Tortuosity, τ, is a geometric parameter describing physical complexity of ionic or mass transport inside the porous electrode network [30]. It is defined as the ratio of the shortest path between two endpoints through the porous compact to its Euclidean length here with τ>1. The tortuosity can be obtained experimentally via direct measurements of diffusion fluxes throughout a porous membrane using diffusion cells, i.e., the Graham and Wicke-Kallenbach cells [31], and through numerical algorithms such as fast marching method [32], Lattice-Boltzmann method (LBM) [33], random walk method [33,34,35,36], pore centroid method [14,30,37] and recently path searching algorithms [38] and A-star algorithm [39]. Another option is to directly model the transport processes through the reconstructed microstructure applying methods of computational fluid dynamics [30,40,41]. The tortuosity of the porous functional layer, as a microstructural characteristic, is treated independently of the operating conditions or diffusing fluid. Due to the significant variations in the tortuosity values obtained by different methods the most suitable tortuosity calculation method for determination of porous structure impact on transfer of fluid species is still under debates.

Moreover, there are limitations due to computational efficiency of the methods applied to calculate the tortuosity from microstructure images, i.e., random walk and LBM, such as in the work implemented by Hlushkou et al. [36] random walk particle-tracking method needs approximately 7000 core-hours to obtain the long-time asymptotes of the D(t) curves for the determination of the effective diffusion coefficients, $D_{eff}$. To describe a pore network for a packing of monosize spheres Voronoi tessellation and random walk method were utilized by Richard et al. [42], the results were well correlated with an experimental data obtained on the transverse diffusion. Semeykina et al. [43] applied Voronoi diagram for tortuosity calculations to define the diffusion coefficient for the optimal catalyst texture via Dijkstra’s algorithm to find the shortest path and demonstrated the computational efficiency as well as relative simplicity in the implementation. However, the lack of appropriately reflected relationships between the compact morphology parameters and transport properties, in particular effective diffusivity, has led our study to aim at disclosing the relationship between Voronoi parameters and tortuosity which will allow anticipating optimum solutions for particle mixture characteristics in particular particle size ratio and mixture composition. In order to investigate the role of particle size ratio and mixture composition in this work ternary mixtures are considered. The addition of a third type of particle to the binary packing results in a broader range of particle size distribution parameters allowing producing number of different cases and number of particle size ratio. It is known that the particle size ratio greatly affects the voidage and thus tortuosity values [44]. Additionally, even numerous studies have been already dedicated to analysis of binary systems [45,46,47,48], there are limited numerical studies on the structural properties of ternary mixtures of spheres [44,49,50,51].

Therefore, the main goal of this work is to study the impact of the packing structure of particle compact on the transport properties in the compact through the calculation of tortuosity and to investigate the relation between tortuosity and the compact packing characteristics, which in turn are correlated to the properties of ternary particle mixture forming the compact.

In the present work, the complex structure of compact, geometrically similar to the actual porous electrode, is reproduced as a random packing of polydisperse spheres by DEM. The radical Voronoi tessellation, which is an extension of the original and well established Voronoi tessellation, is applied here to characterize the packings of multisized particles. The ordinary Voronoi tessellation deals only with the packing of uniform or monosized spheres. Thus, it has been extended to account for a different particle size. There are several approaches such as radical tessellation [52] and navigation map introduced by [53] and further developed by Richard [45,46] to describe the packing of multisized particle mixtures. In the ordinary Voronoi method the neighboring particles are separated by bisecting plane, whereas in the radical tessellation they are separated by radical plane, which is a set of points located in equal tangent distance to the particles, and in the navigation map the Voronoi cell faces consist of the sets of points equally distant to the surface of the particles.

The radical tessellation method was successfully utilized for the description of the packing of polydisperse sphere mixtures in many studies [47,48,54,55,56]. The generation of radical tessellation in the present work is facilitated by an implementation of the algorithms described by Rycroft [57]. This open-source software is also compatible with the DEM results which accelerated analysis of multiple compact structures.

The topological and metric properties of radical Voronoi polyhedron are quantified and analyzed. The tortuosity is simulated using Dijkstra’s algorithm on radical Voronoi tessellation. Then, the parameters of radical Voronoi cells are related to tortuosity. The procedure for generating the particle compacts is detailed in Section 2, Section 3 investigates the packing structure of each particle compact, and discusses the relationship between the tortuosity values and radical Voronoi parameters. Finally, the conclusions are drawn in Section 4.

## 2. Materials and Methods

### 2.1. Methodology

**Figure 1:**
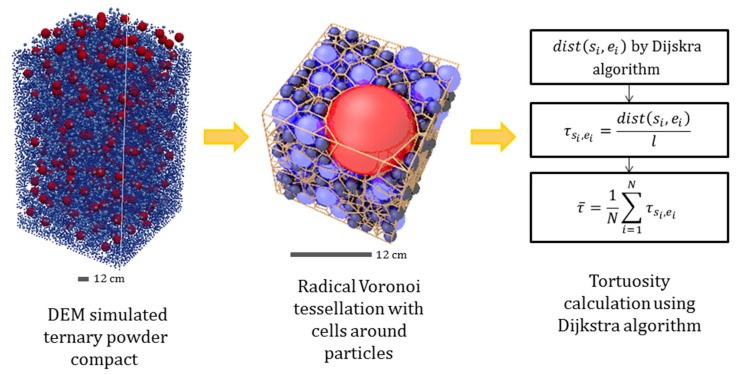
Schematic diagram for calculation of tortuosity. Here, $dist\left(s_{i},\;e_{i}\right)$ is the length of the shortest path between pairs of starting $s_{i}$ and ending $e_{i}$ vertices lying on the opposite faces of the sample box. l is the Euclidean distance. $\tau_{s_{i},e_{i}}$ is the tortuosity calculated for each vertex pairs $s_{i}$ and $e_{i}$. $\bar{\tau }$ is the mean tortuosity, N is the total number of tortuosity values.

Tortuosity assessment of particle compacts was conducted in three primary steps, as illustrated in Figure 1, including: (1) generation of compacts made of ternary mixtures of spherical particles having different compositions and particle size ratios using DEM; (2) construction of radical Voronoi tessellation of particle compacts; (3) calculation of tortuosity for every compact applying Dijkstra’s shortest path algorithm on constructed Voronoi tessellation. Following that, Voronoi cell parameter effects on the calculated tortuosity were studied.

### 2.2. DEM Simulations

Originally developed by Cundall and Strack [58], the discrete element method (DEM) was used here to generate the particle compacts. To model different electrode structures, fifteen ternary powder compacts with varying fractions of small(s), medium (m) and large (L) randomly located spherical particles were generated using an open-source software package LIGGGHTS [59]. A 3D box of size 0.8 m × 0.8 m × 3 m was used as a domain to carry out the simulations. The periodic boundary conditions (pp) were imposed along the x and y axes and the fixed boundary conditions (ff) were implemented along the z axis. The periodic boundaries were used to minimize the wall effect during the particle compaction process.

First, 5000–70,000 particles comprising the ternary mixture of the specified composition were distributed randomly throughout the 3D domain. Next, particle compacts have been formed under the gravitational force (9.81 m/s^2^) for 7,000,000 time steps (Δt = 5 × 10^−7^ s). Table 1 shows the mechanical properties of particles. As summarized in Table 2, the ternary mixtures of three size ratios each having seven differing volume fractions were modeled in the present study. It should be noted that in order to perform DEM simulations efficiently, the particles of larger sizes compared to that of real electrodes were used in simulations, however, the packing values of generated compacts were relatively close to the packing values of real structures (about 0.7) [27,60]. Additionally, the difference between the large and small size components was set to account for the influence of various additives on the electrode microstructure. The value of Young’s modulus was assigned to be the same as in [61] and three magnitudes less compared to that of the real graphite electrode. This calibration was also made to improve the simulation time [62,63]. The number of the simulated particles in a compact was large enough to represent the packing of material in the electrode and allows parametrically study compacts as electrodes microstructure.

To achieve the stability of the system in the DEM simulations we applied a small time step Δt = 5 × 10^−7^ s and simulation of each sample were run for long time in order to allow the particles to settle down and then to reach an equilibrium state, moreover we continued our simulations until the kinetic energy is essentially zero. Within a reasonable timeframe it is not possible to repeat DEM simulations to generate packing compact replicas. Although the packings are randomly generated in the DEM simulations, and the position of the particles may not be identical in replicas, in a study by Ramírez-Aragón et al. [64] it was confirmed that this difference had a negligible impact on the results.

### 2.3. Radical Voronoi Tessellation

To carry out the analysis of packing structure of compacts, a cube of size 0.2 m × 0.2 m × 0.2 m was cut out from the every obtained compact with the purpose of elimination of possible irregularities close to the top and bottom compact boundaries. In our preliminary calculations we compared radical Voronoi parameters of three randomly located cubes within the compact generated in the same DEM simulation. The results of this comparison prove that sample cube packing structure are similar to each other. Thus, for further tortuosity calculations we used only one cube per one DEM simulation.

**Figure 2:**
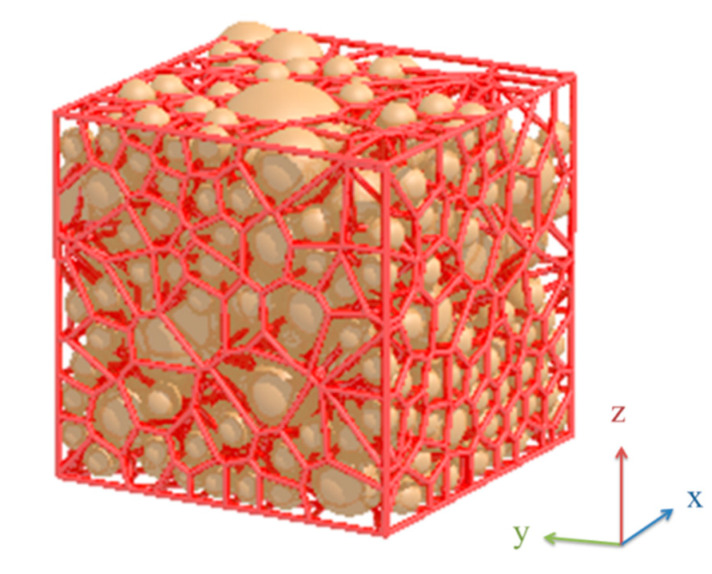
Illustration of typical radical Voronoi tessellation for ternary mixture with volume fraction of small, medium and large particles equal to 25%:25%:50% and particle size ratio 1:2:4.

The radical Voronoi diagram was used to represent the arrangements of particles in the compact and, thus, the porous space among particles. The diagram is constructed from a set of polyhedron cells, as shown in Figure 2. Properties of the system of cells are as follows: (1) every pair of cells have one common face; (2) every three adjacent cells have one common edge; (3) every adjacent four cells have one common vertex. An open-source software package Voro++ [57] was used for creation of the radical Voronoi tessellation on the compact samples. In addition to breaking up particle compact samples into polyhedron radical Voronoi cells, the cell parameters including area per face of the radical Voronoi cell and the edge lengths of the radical Voronoi cell were computed using Voro++ software.

### 2.4. Tortuosity Calculations

Tortuosity values are used in the calculation of the effective diffusion coefficient $D_{eff}$. The correlation between $D_{eff}$ and tortuosity is as follows [65]:

(1) $$D_{eff}=D_{bulk}\;\times \;\frac{\varepsilon }{\tau }$$

where $D_{bulk}$ stands for the combination of Knudsen and molecular diffusivities for a straight pore, ε is the corresponding network porosity and τ is the tortuosity.

To calculate the tortuosity of particle compact samples we have used their Voronoi diagrams. For this purpose, the shortest path through the network of radical Voronoi cell edges was estimated using the Dijkstra’s shortest path algorithm [66]. Boundary vertices for tortuosity analysis were generated on the edge intersections with the plane of each face of the sample box. The length of the shortest path, $dist\left(s_{i},\;e_{i}\right)$ was calculated between every boundary vertex on one face of the sample box, $s_{i}$, and all boundary vertices on the opposite face of the sample box, $e_{i}$, separately in three directions x, y and z. Consequently, there are three different set of data for x, y and z directions.

Dijkstra’s algorithm includes the following steps:

- All nodes, i.e., Voronoi cell vertices, are marked as unvisited and a set of unvisited nodes are created.
- A preliminary distance value is set to infinity for all nodes except the starting node for which it should be equal to zero. The starting node is set as a current node [67].
- The preliminary distances are calculated through the current node to all of its unvisited neighbors. The preliminary distance calculated lately is compared to the already assigned current value and then the smaller one is chosen and assigned to the node.
- When consideration of all the unvisited current node neighbors is completed, the current node is signed as a visited node and it is withdrawn from the list of unvisited nodes. Once the node appears on the visited list, the node will not be checked anymore.
- The algorithm is complete after the destination node is marked as the visited one. Otherwise, the next current node is chosen from the list of unvisited nodes as a node with the smallest preliminary distance and the cycle is repeated from the step 3.

Then, the tortuosity was calculated for each vertex pairs $s_{i}$ and $e_{i}$ as

(2) $$\tau_{s_{i},e_{i}}=\frac{dist\left(s_{i},\;e_{i}\right)}{l}$$

where l is the Euclidean distance.

Following that, the tortuosity distribution curves were built. The central trend in the tortuosity value population is represented by average values such as mode, median and mean values. The mode, $\tau_{m}$, is the most common value in the distribution and it is the tortuosity value corresponding to the maximum frequency. The median, $\tau_{50}$, is the tortuosity value at which the distribution is divided into two equal parts and the cumulative distribution is equal to 50%. The mean, $\bar{\tau }$, is the ratio of sum of values of tortuosity to the total number of the values, N:

(3) $$\bar{\tau }=\frac{1}{N}\overset{N}{\underset{i=1}{\sum }}\tau_{i}$$

The width of the tortuosity distribution curve is described by the span value which is defined as

(4) $$s=\frac{\tau_{90}-\tau_{10}}{\tau_{50}}$$

where $\tau_{10}$ and $\tau_{90}$ are the tortuosities corresponding to the 10% and 90% on the cumulative distribution curve, respectively.

The tortuosity simulations were performed in Matlab. The lengths of Voronoi edges were calculated in Matlab, as well, using the coordinates of vertices and the information about the existence of an edge between two vertices.

## 3. Results and Discussion

**Figure 3:**
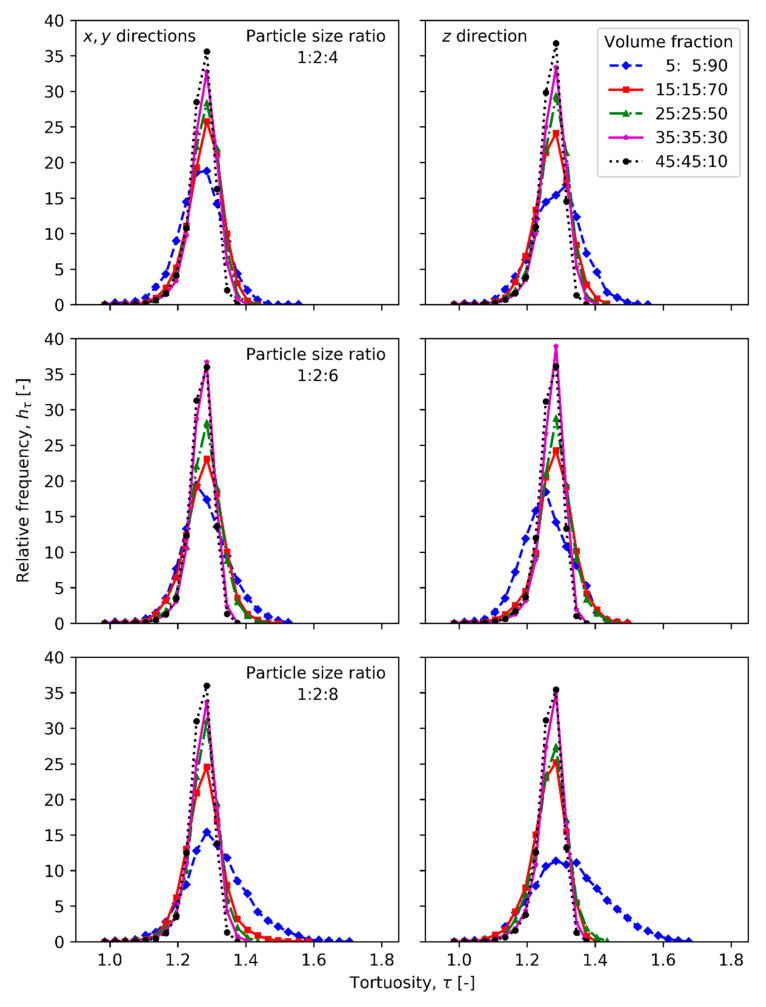
Relative frequency of tortuosity of ternary mixtures having particle size ratio of 1:2:4, 1:2:6 and 1:2:8 with different volume fractions evaluated in x, y and z directions.

**Figure 4:**
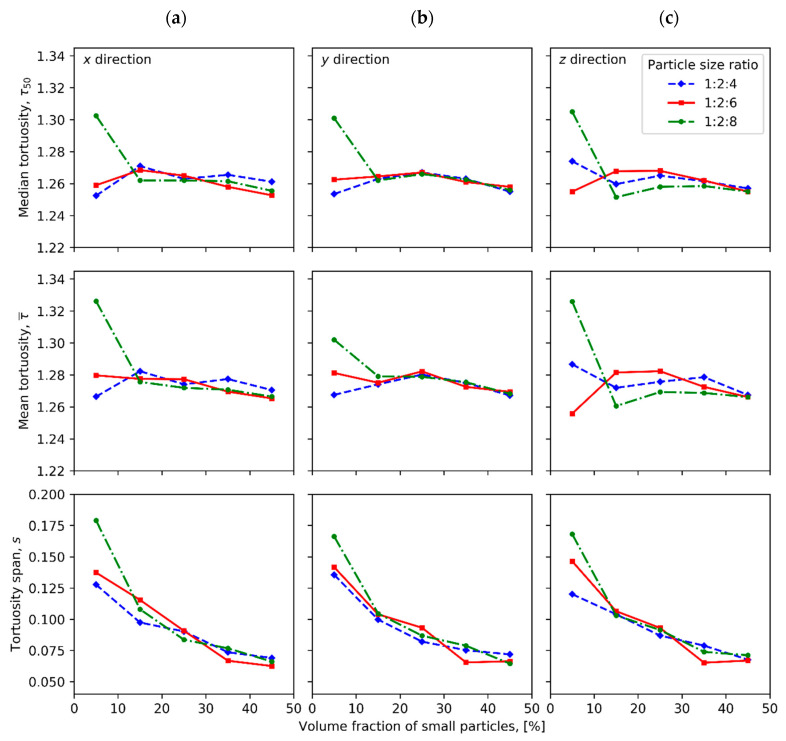
Median, mean and span of tortuosity distribution of compacts of various volume fractions of small particles measured in (**a**) x, (**b**) y and (**c**) z directions.

**Figure 5:**
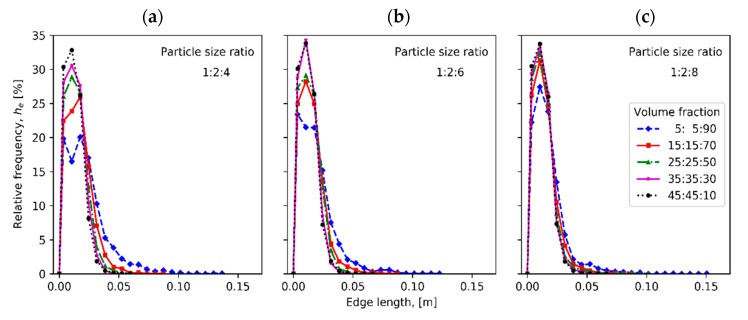
Relative frequency of edge length of radical Voronoi cells for samples having different volume fractions and three instances of particle size ratio: (**a**) 1:2:4, (**b**) 1:2:6 and (**c**) 1:2:8.

**Figure 6:**
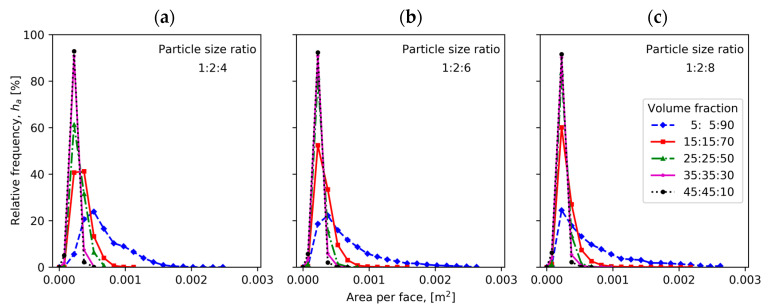
Relative frequency of area per face of radical Voronoi cells for samples having different volume fractions and three instances of particle size ratio: (**a**) 1:2:4, (**b**) 1:2:6 and (**c**) 1:2:8.

Figure 3 compares tortuosity measured in x, y and z directions for compacts of different particle fractions with particle size ratios 1:2:4, 1:2:6 and 1:2:8. Figure 4 illustrates the mean, median and span values of tortuosity distribution for the ternary mixture compacts. The relative frequencies of edge lengths and of area per face of radical Voronoi polyhedrons are plotted in Figure 5 and Figure 6, respectively.

### 3.1. Tortuosity Dependence on Parameters of Ternary Mixture

The decrease of tortuosity relative frequency peaks from around 35% to nearly 17% is observed in all compact directions with the growth of volume fraction of the large particles from 10% to 90% for compacts made of ternary mixtures having the particle size ratio of 1:2:4, as shown in Figure 3. The greater the amount of large particles, the larger the maximum tortuosity value in the distribution (1.375 for 10%and 1.555 for 90%, respectively).

In a similar manner with the previous case, the tortuosity distribution peaks for the samples with particle size ratios of 1:2:6 drop from approximately 36% to 18% with the increase of large particles in the mixture, with minor exceptions, as illustrated in Figure 3. The distributions are wider for samples with greater fraction of large particles, and the largest tortuosity value being equal to 1.375 and 1.525 for samples with 10% and 90% of large particles, respectively.

The tortuosity distribution peaks for the samples with particle size ratio of 1:2:8 also decrease as in the former cases by increasing the volume fraction of large particles from 36% to 11%, as depicted in Figure 3. In this case, the maximum tortuosity value in the distribution remains at τ = 1.375 for the sample with volume fraction of 45%:45%:10% and continued to much larger value of 1.705 than in the previous cases for the sample with volume fraction of 5%:5%:90%. It is easily noticeable that the sample with volume fraction of 5%:5%:90% has the pronounced right-skewed tortuosity distributions in all directions.

As can be seen from the average value graphs (Figure 4), the median tortuosity generally fluctuates around 1.260, and the mean tortuosity fluctuates in the same manner around 1.270, while the mode has a plateau at 1.285 for all cases of particle ratios and volume fractions, with some exceptions for the samples with volume fraction of 5%:5%:90%. It is worth to note that the arrangement of the average values such as median < mean < mode confirms that the distribution curves of these samples are skewed to the right. The slightly larger average values of median tortuosity at around 1.3 and mean tortuosity at near to 1.32 for mixtures with volume fraction of 5%:5%:90% and particle size ratio of 1:2:8 result to more asymmetric and positively skewed tortuosity distribution. Deviating from the common trend mode values for the samples with volume fraction of 5%:5%:90% are observed for the sample with particle size ratio of 1:2:6 having slightly different mode values of 1.255 in x- and y- and 1.239 in z-directions and the sample with particle size ratio of 1:2:4 having only one deviation in z-direction being equal to 1.315. With the decrease in the volume fraction of large particle from 90% to 10%, the span of tortuosity distribution falls from around 0.127 to nearly 0.069 for the samples with particle size ratio of 1:2:4, from approximately 0.14 to barely 0.065 for the samples with particle size ratio of 1:2:6, and from around 0.17 to nearly 0.067 for the samples with particle size ratio of 1:2:8, as shown in Figure 4.

Our calculated values of tortuosity are in the range of those obtained by experimental methods for a ternary mixture from 1.1547 to 1.58 [68,69].

### 3.2. Dependence of Radical Voronoi Parameters on Ternary Mixture Parameters

Figure 5 demonstrates that an increase of the volume fraction of large particles in the mixture leads to the lowering of peak values in the relative frequency of radical Voronoi edge length curves as in the tortuosity distribution curves. With the increase of large particles in number and size, the longer radical Voronoi edges appear. Although the distribution of radical Voronoi edge length for the sample with volume fraction of 5%:5%:90% and particle size ratio of 1:2:8 lasts to the longest edges than in other cases, its peak is sharper.

With the increase of large particle volume fractions in the mixture, the peaks of the area per face relative frequency of radical Voronoi cells decrease for samples of all particle size ratios, while the ranges of the distributions widen, as illustrated in Figure 6. Moreover, more positively skewed distribution is noticeable with the increase of large particle sizes for fraction 5%:5%:90%, while the mode area per face shifts to the left from 0.000525 m for the sample with particle size ratio of 1:2:4 to 0.000225 m for the sample with particle size ratio of 1:2:8.

Taking all the above into consideration, the dependency of tortuosity on radical Voronoi cell parameters can be summarized in the following way. The appearance of larger faces and longer edges of radical Voronoi cells results in increase of frequency of large tortuosity values, whilst smaller faces and shorter edges lead to smaller and more uniform tortuosity values with narrower distribution. Consequently, considering the inverse relation between the diffusion coefficient and the tortuosity by Equation (1), the smaller faces and shorter edges of radical Voronoi cells corresponding to the higher volume fraction of small and medium particles contribute to an improved diffusivity. Moreover, there are experimental studies confirming that the addition of small particles such as nano-additives improves improve electron transfer in the electrode, and thus positively influence on the kinetic and transport properties [70]. The parametric study described in the present study will subsequently allow one to select the composition of particle mixture in order to form the sufficiently packed compact having enhanced diffusion properties.

The bonding effect between particles is important to consider for porous materials under compaction and in the production. The present DEM simulation can be enhanced in future by adding the bonding effects to particle-particle interactions [71] in order to model processes in the production steps and obtain range of mechanical properties of the compacts.

## 4. Conclusions

The present study reflects one of the first attempts at the tortuosity analysis of modeled porous compacts of ternary mixtures of spherical particles using the radical Voronoi tessellation. First of all, we have simulated ternary powder compacts with three size ratios of small, medium and large spherical particles and each having five different volume fractions of particles using DEM. Then, the radical Voronoi tessellation has been constructed for obtained compacts. Finally, the tortuosity distributions were simulated in every axis direction of the compact applying the Dijkstra’s shortest path algorithm on radical Voronoi diagrams.

Based on the results of our simulations, the correlations were established between the compact tortuosity and the particle mixture properties including volume fractions and particle size ratio. With the increase in the small and medium particle volume fractions from 5% to 45%, the tortuosity distribution peaks tend to grow and become sharper, narrowing the tortuosity distribution ranges, for the every case of particle size ratio. At the same time, the increase in the large particle volume fraction from 10% to 90% leads to shorter, less sharp tortuosity distribution peaks and wider tortuosity distribution ranges. The changes in particle size ratio do not result in the significant changes in the tortuosity distribution except the samples with volume fraction of 5%:5%:90%. In these samples the increase of the size of large particles results in more positively skewed and wider distributions.

The relationships between tortuosity and radical Voronoi cell parameters were analyzed in the present study. The increase of frequency of larger tortuosity values was associated with larger faces and longer edges of radical Voronoi cells, while smaller tortuosity values having narrower distribution were related to the smaller faces and shorter edges. Therefore, given that diffusion coefficient and the tortuosity are inversely related, the higher volume fractions of small and medium particles contribute to an improved diffusion coefficient due to smaller faces and shorter edges of radical Voronoi cells. Thus, the results of present research will help to design the composition of particle mixture in order to manufacture the well-packed particle compact having enhanced diffusion properties.

## Figures and Tables

**Table 1:** Mechanical properties of particles.

Mechanical Properties	
Particle density, *ρ* (kg/m^3^)	2500
Poisson ratio, *σ*	0.45
Young’s modulus, *Y* (N/m^2^)	5 × 10^6^

**Table 2:** Ternary mixture composition and particle size ratios.

Particle Radii (cm) 1:2:4	**#**	**Volume Fraction (%)**			Particle Radii (cm) 1:2:6	**#**	**Volume Fraction (%)**			Particle Radii (cm) 1:2:8	**#**	**Volume Fraction (%)**		
		**s**	**m**	**L**			**s**	**m**	**L**			**s**	**m**	**L**
	1	5	5	90		6	5	5	90		11	5	5	90
	2	15	15	70		7	15	15	70		12	15	15	70
	3	25	25	50		8	25	25	50		13	25	25	50
	4	35	35	30		9	35	35	30		14	35	35	30
	5	45	45	10		10	45	45	10		15	45	45	10

## References

[1] El Kharbachi A., Zavorotynska O., Latroche M., Cuevas F., Yartys V., Fichtner M. (2020). Exploits, advances and challenges benefiting beyond Li-ion battery technologies. J. Alloys Compd..

[2] Wang Y., Fu X., Zheng M., Zhong W., Cao G. (2018). Strategies for Building Robust Traffic Networks in Advanced Energy Storage Devices: A Focus on Composite Electrodes. Adv. Mater..

[3] Sun Y., Liu N., Cui Y. (2016). Promises and challenges of nanomaterials for lithium-based rechargeable batteries. Nat. Energy.

[4] Ellis B.L., Nazar L.F. (2012). Sodium and sodium-ion energy storage batteries. Curr. Opin. Solid State Mater. Sci..

[5] Ma L., Hendrickson K.E., Wei S., Archer L.A. (2015). Nanomaterials: Science and applications in the lithium-sulfur battery. Nano Today.

[6] Zhao Y., Zhang Y., Bakenov Z., Chen P. (2013). Electrochemical performance of lithium gel polymer battery with nanostructured sulfur/carbon composite cathode. Solid State Ion..

[7] Wang J., Chen Y., Qi L. (2011). The development of silicon nanocomposite materials for Li-Ion secondary batteries. Open Mater. Sci. J..

[8] Mukherjee R., Krishnan R., Lu T.-M., Koratkar N. (2012). Nanostructured electrodes for high-power lithium ion batteries. Nano Energy.

[9] Yan Z., Hara S., Shikazono N. (2017). Effect of powder morphology on the microstructural characteristics of La_0.6_Sr_0.4_Co_0.2_Fe_0.8_O_3_ cathode: A Kinetic Monte Carlo investigation. Int. J. Hydrogen Energy.

[10] Zhang D., Bertei A., Tariq F. (2019). Progress in 3D electrode microstructure modelling for fuel cells and batteries: Transport and electrochemical performance. Prog. Energy.

[11] Lim C., Yan B., Kang H., Song Z., Lee W.C., De Andrade V., De Carlo F., Yin L., Kim Y., Zhu L. (2016). Analysis of geometric and electrochemical characteristics of lithium cobalt oxide electrode with different packing densities. J. Power Sources.

[12] Wilson J.R., Cronin J.S., Barnett S.A., Harris S.J. (2011). Measurement of three-dimensional microstructure in a LiCoO_2_ positive electrode. J. Power Sources.

[13] Chung D.-W., Shearing P.R., Brandon N.P., Harris S.J., Garcia R.E. (2014). Particle Size Polydispersity in Li-Ion Batteries. J. Electrochem. Soc..

[14] Cooper S.J., Eastwood D.S., Gelb J., Damblanc G., Brett D.J.L., Bradley R.S., Withers P.J., Lee P.D., Marquis A.J., Brandon N.P. (2014). Image based modelling of microstructural heterogeneity in LiFePO_4_ electrodes for Li-ion batteries. J. Power Sources.

[15] Miranda D., Gören A., Costa C.M., Silva M.M., Almeida A.M., Lanceros-Méndez S. (2019). Theoretical simulation of the optimal relation between active material, binder and conductive additive for lithium-ion battery cathodes. Energy.

[16] Doyle M., Newman J. (1995). Modeling the performance of rechargeable lithium-based cells: Design correlations for limiting cases. J. Power Sources.

[17] Martínez-Rosas E., Vasquez-Medrano R., Flores-Tlacuahuac A. (2011). Modeling and simulation of lithium-ion batteries. Comput. Chem. Eng..

[18] Shah A.A., Luo K.H., Ralph T.R., Walsh F.C. (2011). Recent trends and developments in polymer electrolyte membrane fuel cell modelling. Electrochim. Acta.

[19] Shah A.A., Watt-Smith M.J., Walsh F.C. (2008). A dynamic performance model for redox-flow batteries involving soluble species. Electrochim. Acta.

[20] Zhu Y., Pham H., Park J. (2019). A New Aspect of the Li Diffusion Enhancement Mechanism of Ultrathin Coating Layer on Electrode Materials. ACS Appl. Mater. Interfaces.

[21] Wang Z., Ma J., Zhang L. (2017). Finite Element Thermal Model and Simulation for a Cylindrical Li-Ion Battery. IEEE Access.

[22] Tang M., Albertus P., Newman J. (2009). Two-Dimensional Modeling of Lithium Deposition during Cell Charging. J. Electrochem. Soc..

[23] Wu J., Srinivasan V., Xu J., Wang C.Y. (2002). Newton-Krylov-Multigrid Algorithms for Battery Simulation. J. Electrochem. Soc..

[24] Chuang C., Singh D., Kenesei P., Almer J., Hryn J., Huff R. (2018). Application of X-ray computed tomography for the characterization of graphite morphology in compact-graphite iron. Mater. Charact..

[25] Faÿ-Gomord O., Soete J., Davy C.A., Janssens N., Troadec D., Cazaux F., Caline B., Swennen R. (2017). Tight chalk: Characterization of the 3D pore network by FIB-SEM, towards the understanding of fluid transport. J. Pet. Sci. Eng..

[26] Giménez C.S., Schilde C., Froböse L., Ivanov S., Kwade A. (2020). Mechanical, Electrical, and Ionic Behavior of Lithium-Ion Battery Electrodes via Discrete Element Method Simulations. Energy Technol..

[27] Giménez C.S., Finke B., Nowak C., Schilde C., Kwade A. (2018). Structural and mechanical characterization of lithium-ion battery electrodes via DEM simulations. Adv. Powder Technol..

[28] Delaney G.W., Di Matteo T., Aste T. (2010). Combining tomographic imaging and DEM simulations to investigate the structure of experimental sphere packings. Soft Matter.

[29] Yan Z., Hara S., Shikazono N. (2018). Towards a realistic prediction of sintering of solid oxide fuel cell electrodes: From tomography to discrete element and kinetic Monte Carlo simulations. Scr. Mater..

[30] Kehrwald D., Shearing P.R., Brandon N.P., Sinha P.K., Harris S.J. (2011). Local Tortuosity Inhomogeneities in a Lithium Battery Composite Electrode. J. Electrochem. Soc..

[31] Soukup K., Schneider P., Šolcová O. (2008). Comparison of Wicke-Kallenbach and Graham’s diffusion cells for obtaining transport characteristics of porous solids. Chem. Eng. Sci..

[32] Jørgensen P.S., Hansen K.V., Larsen R., Bowen J.R. (2011). Geometrical characterization of interconnected phase networks in three dimensions. J. Microsc..

[33] Iwai H., Shikazono N., Matsui T., Teshima H., Kishimoto M., Kishida R., Hayashi D., Matsuzaki K., Kanno D., Saito M. (2010). Quantification of SOFC anode microstructure based on dual beam FIB-SEM technique. J. Power Sources.

[34] Kishimoto M., Iwai H., Saito M., Yoshida H. (2019). Quantitative Evaluation of Transport Properties of SOFC Porous Anode by Random Walk Process. ECS Trans..

[35] Chueh C.C., Bertei A., Pharoah J.G., Nicolella C. (2014). Effective conductivity in random porous media with convex and non-convex porosity. Int. J. Heat Mass Transf..

[36] Hlushkou D., Svidrytski A., Tallarek U. (2017). Tracer-Size-Dependent Pore Space Accessibility and Long-Time Diffusion Coefficient in Amorphous, Mesoporous Silica. J. Phys. Chem. C.

[37] Gostovic D., Smith J.R., Kundinger D.P., Jones K.S., Wachsman E.D. (2007). Three-dimensional reconstruction of porous LSCF cathodes. Electrochem. Solid-State Lett..

[38] Sobieski W. (2016). The use of Path Tracking Method for determining the tortuosity field in a porous bed. Granul. Matter.

[39] Sobieski W. (2020). Calculating the Binary Tortuosity in DEM-Generated Granular Beds. Processes.

[40] Chung D.W., Ebner M., Ely D.R., Wood V., García R.E. (2013). Validity of the Bruggeman relation for porous electrodes. Model. Simul. Mater. Sci. Eng..

[41] Trogadas P., Taiwo O.O., Tjaden B., Neville T.P., Yun S., Parrondo J., Ramani V., Coppens M.-O., Brett D.J.L., Shearing P.R. (2014). X-ray micro-tomography as a diagnostic tool for the electrode degradation in vanadium redox flow batteries. Electrochem. Commun..

[42] Richard P., Oger L., Lemaître J., Samson L., Medvedev N.N. (1999). Application of the Voronoï tessellation to study transport and segregation of grains inside 2D and 3D packings of spheres. Granul. Matter.

[43] Semeykina V.S., Malkovich E.G., Bazaikin Y.V., Lysikov A.I., Parkhomchuk E.V. (2018). Optimal catalyst texture in macromolecule conversion: A computational and experimental study. Chem. Eng. Sci..

[44] Ng T.T., Ge L. (2020). Packing void ratios of very dense ternary mixtures of similar ellipsoids. Granul. Matter.

[45] Richard P., Oger L., Troadec J.P., Gervois A. (1998). Tessellation of binary assemblies of spheres. Phys. A Stat. Mech. Appl..

[46] Richard P., Oger L., Troadec J.P., Gervois A. (2001). A model of binary assemblies of spheres. Eur. Phys. J. E.

[47] Park J., Shibutani Y. (2007). Weighted Voronoi tessellation technique for internal structure of metallic glasses. Intermetallics.

[48] Gervois A., Oger L., Troadec J.P. (2004). Random cuts in binary mixtures of spheres. Phys. Rev. E.

[49] Wiącek J., Stasiak M. (2018). Effect of the particle size ratio on the structural properties of granular mixtures with discrete particle size distribution. Granul. Matter.

[50] Wiącek J., Stasiak M., Kafashan J. (2020). Structural and Micromechanical Properties of Ternary Granular Packings: Effect of Particle Size Ratio and Number Fraction of Particle Size Classes. Materials.

[51] Yi L.Y., Dong K.J., Zou R.P., Yu A.B. (2011). Coordination number of the packing of ternary mixture of spheres: DEM simulations versus measurements. Ind. Eng. Chem. Res..

[52] Gellatly B.J., Finney J.L. (1982). Characterisation of models of multicomponent amorphous metals: The radical alternative to the Voronoi polyhedron. J. Non Cryst. Solids.

[53] Medvedev N.N. (1994). Application of the Voronoi-Delone method to description of structure of intersphere space in polydisperse systems. Doklady Physical Chemistry.

[54] Yi L.Y., Dong K.J., Zou R.P., Yu A.B. (2012). Radical tessellation of the packing of ternary mixtures of spheres. Powder Technol..

[55] Dong K., Wang C., Yu A. (2016). Voronoi analysis of the packings of non-spherical particles. Chem. Eng. Sci..

[56] Chen L., Wang C., Moscardini M., Kamlah M., Liu S. (2019). A DEM-based heat transfer model for the evaluation of effective thermal conductivity of packed beds filled with stagnant fluid: Thermal contact theory and numerical simulation. Int. J. Heat Mass Transf..

[57] Rycroft C. (2009). Voro++: A Three-Dimensional Voronoi Cell Library in C++.

[58] Cundall P.A., Strack O.D.L. (1979). A discrete numerical model for granular assemblies. Geotechnique.

[59] LIGGGHTS^®^-PUBLIC Documentation, Version 3.X, © Copyright 2016, DCS Computing GmbH, JKU Linz and Sandia Corporation. https://www.cfdem.com/media/DEM/docu/Manual.html.

[60] Lotfabad E.M., Ding J., Cui K., Kohandehghan A., Kalisvaart W.P., Hazelton M., Mitlin D. (2014). High-density sodium and lithium ion battery anodes from banana peels. ACS Nano.

[61] Akhmetov Z., Boribayeva A., Berkinova Z., Yermukhambetova A., Golman B. (2019). Microstructural features of ternary powder compacts. Chem. Eng. Trans..

[62] Lommen S., Schott D., Lodewijks G. (2014). DEM speedup: Stiffness effects on behavior of bulk material. Particuology.

[63] Lommen S., Mohajeri M., Lodewijks G., Schott D. (2019). DEM particle upscaling for large-scale bulk handling equipment and material interaction. Powder Technol..

[64] Ramírez-Aragón C., Ordieres-Meré J., Alba-Elías F., González-Marcos A. (2018). Comparison of Cohesive Models in EDEM and LIGGGHTS for Simulating Powder Compaction. Materials.

[65] Tjaden B., Brett D.J., Shearing P.R. (2018). Tortuosity in electrochemical devices: A review of calculation approaches. Int. Mater. Rev..

[66] Dijkstra E. (1959). A note on two problems in connexion with graphs. Numer. Math..

[67] Gass S.I., Fu M.C. (2013). Encyclopedia of Operations Research and Management Science.

[68] Mota M., Teixeira J., Yelshin A. (1999). Image analysis of packed beds of spherical particles of different sizes. Sep. Purif. Technol..

[69] Dias R.P., Mota M., Teixeira J.A., Yelshin A. (2005). Study of ternary glass spherical particle beds: Porosity, tortuosity, and permeability. Filtration.

[70] Ahamad S., Gupta A. (2019). A systematic study of kinetics in mesocarbonmicrobeads anodes in presence of nano-conductive additives. Electrochim. Acta.

[71] Potyondy D.O., Cundall P.A. (2004). A bonded-particle model for rock. Int. J. Rock Mech. Min. Sci..

